# Association between School Achievement and Tobacco Susceptibility among US Adolescents: Ethnic Differences

**DOI:** 10.3390/children10020327

**Published:** 2023-02-09

**Authors:** Edward Adinkrah, Babak Najand, Angela Young-Brinn, Samrand Salimi

**Affiliations:** 1 Department of Family Medicine, Charles R. Drew University of Medicine and Science, Los Angeles, CA 90059, USA; 2 Marginalization-Related Diminished Returns, Los Angeles, CA 90059, USA; 3 Vian Clinic, Tehran 1911915398, Iran

**Keywords:** adolescents, ethnic groups, academic achievement, risk behavior, tobacco use susceptibility

## Abstract

Background: Although risky behaviors such as educational problems and tobacco use tend to co-occur, these associations may vary across diverse ethnic groups, in part because ethnic minorities tend to reside in worse neighborhoods and tend to attend worse schools than Non-Latino White adolescents. Aim: To compare the association between baseline school achievement (student grades) and subsequent tobacco use susceptibility (openness to smoke in future) by ethnicity, we compared African American, Latino, and Non-Latino White adolescents in the US over a four-year period. Methods: This longitudinal study followed 3636 adolescents who were never smokers at baseline for a period of four years. Baseline and four-year data of the Population Assessment of Tobacco and Health (PATH) study were used for this analysis. All participants were 12 to 17 years old at baseline and were either Non-Latino White (Majority), African American (Minority), or Latino (Minority). The outcome was a tobacco use susceptibility score at wave 4 which was defined as openness to use tobacco in the future, measured at year four. The predictor was school achievement at wave 1, measured as grades from F to A+. The moderator was ethnicity (African American, Latino, Non-Latino White), and covariates were age, gender, parental education, and family structure. Results: Our linear regressions in the pooled sample showed an inverse association between baseline school achievement and subsequent tobacco use susceptibility four years later. However, this inverse association was weaker for ethnic minorities than for Non-Latino White adolescents, as documented by interaction effects between ethnic minority status and baseline school grades. Conclusion: Higher educational success better correlates with lower tobacco use susceptibility of non-Latino White than African American and Latino adolescents, which may reflect some tobacco use susceptibility of Latino and African American adolescents with highly educated parents. Future research should investigate how social context such as high-risk school environment, neighborhood risk, peer risk, and other mechanisms increase behavioral risk of educationally successful African American and Latino adolescents.

## 1. Background

Adolescence is a developmental period with an increased risk of problem behaviors such as tobacco use and school dropout. According to the Jesser and Jesser theory [1], problem behaviors tend to covary in adolescents, with some individuals showing none or a low number of problem behaviors and other students showing various types of risks simultaneously [1]. In this view, each risk behavior increases the likelihood of other risk behaviors. This overlap may be due to common causes, confounding, selection, or presence of risk at social network, poor neighborhoods, or poor parenting, that increase risk of undesired behaviors, regardless of their specific type. Thus, some adolescents show a range of problems such as tobacco use, school dropout, aggression, drug use, and even health problems [1].

At the same time, higher educational attainment is associated with lower risk-taking. A growing body of research has shown links between health behaviors and school achievement [2]. Multiple studies have shown significant associations between school achievement and nutrition [3], violence [4], and physical exercise [5]. Research has also shown a link between educational success and low prevalence of smoking [6,7] and alcohol use [8,9]. The protective association between higher school achievement and lower tobacco use susceptibility may be in part due to behavioral and risk profile of peers, or other factors such as parent education, parental engagement, or quality of schools and neighborhoods. Self-selection may also be a mechanism that connects these behaviors [1]. However, as nothing is universal, and as ethnic groups live in different contexts, this clustering of risk may vary by ethnicity. Therefore, it is imperative to explore ethnic variation in the association between school achievement and tobacco use susceptibility in US adolescents [10].

The association between school achievement and health risk, however, differs by ethnicity [11,12,13]. Fuller-Rowell has shown that the association between school achievement and health may be racialized [11,12,13]. Under racism and discrimination, more costs may be involved in the educational success of ethnic minorities than Non-Latino White adolescents [11,12,13]. That means that while this association exists for Non-Latino Whites, the same association may not exist or be weaker for ethnic minority adolescents who, in their social context, may pay a higher level of psychological tax for their educational success, or chronic poverty from childhood [11,12,13]. Fuller-Rowell has conducted multiple studies and showed that the positive association between school achievement and health is weaker or reverse for African American than White adolescents [11,12,13]. His first study investigated ethnic groups in a nationally representative sample of adolescents for comparison of the longitudinal association between academic achievement and social acceptance. They also explored the effects of school context. This study included a total number of 13,570 adolescents who were 15.5 years on average. The study showed that ethnic minority adolescents pay greater social costs with academic success than Non-Latino Whites; however, this is seen in high-achieving schools with a smaller percentage of ethnic minority students [14]. In his second study, 1192 ethnic minorities and 1487 Non-Latino White adolescents participated. Measurements were available at age = 30 and 45. Author analyses focused on age-related changes in fibrinogen, C-reactive protein, and interleukin-6 across ethnic groups. Then, the effects of educational attainment on changes in inflammation were modeled for ethnic minorities and Non-Latino Whites before and after controlling for four blocks of covariates: (a) early life adversity, (b) baseline health and health behaviors, (c) employment and financial measures at baseline and follow-up, and (d) adulthood psychosocial stress. Their first finding was that ethnic minorities had larger increases in fibrinogen over time than Non-Latino Whites. Effects of educational attainment were weaker for ethnic minorities than for Non-Latino Whites, and only 8% of this difference was due to covariates. Analyses yielded consistent results. They concluded that the effects of educational attainment on inflammation levels are stronger for Non-Latino Whites than for ethnic minorities [12].

Highly successful ethnic minorities students are likely to be discriminated against [15,16], attend poor schools [17], and have high risk peers and family members [18]. When they move to schools that have high SES and predominantly Non-Latino White, they become even more discriminated against [19,20]. In addition, education of ethnic minorities in a system designed for Whites is a taxing process [21], given the challenges of ethnic minorities at schools [22]. All these suggest that correlates of education success may vary by ethnicity [11,12,13].

Marginalization-related diminished returns (MDRs) theory suggests that resources and assets may be associated with lower levels of economic, behavioral, developmental, and health outcomes for marginalized and racialized groups than non-Latino White individuals [23,24]. Although most of this literature is generated on SES indicators [25,26,27,28,29,30,31,32], some research also shows that psychological assets such as self-efficacy [33], positive affect [34,35], and happiness [36,37,38] may also generate more health for Non-Latino Whites than ethnic minorities. In easy to control environments, protective assets are probably health protective. In environments that are difficult to control, however, agency may become detrimental. In this view, ethnic minority individuals with high sense of agency may encounter more challenges than others; however, at the same time, the very same environment may foster the success of Non-Latino Whites with similar agency [33,39]. 

Based on the minorities’ diminished returns theory [40,41], and given the well-established literature on educational challenges [22] and discrimination [15,16] between highly ambitious Non-Latino White adolescents who attend better schools [17] at US schools [19,20], this study tested the association between school achievement and tobacco use susceptibility overall and also by ethnicity. Our first hypothesis was that overall, high school achievement is associated with lower tobacco use susceptibility. Our second hypothesis was that this inverse association is weaker for ethnic minorities than Non-Latino White adolescents.

## 2. Methods

### 2.1. Design and Setting

This was a secondary analysis of the Population Assessment of Tobacco and Health (PATH) study. Population Assessment of Tobacco and Health (PATH) is the state-of-the-art study of tobacco and tobacco use susceptibility of adolescents and adults in the US. For the current analysis, we used data from the PATH adolescents subsample.

### 2.2. Sample and Sampling

In the PATH study, participants were selected randomly. Stratified and clustered random samples were selected from all US states. Eligibility was non-institutionalized members of US households. All participants were aged between 12 and 17. Only ethnic minorities and Non-Latino White participants were included in this analysis. 

### 2.3. Variables

Study variables in this analysis included ethnicity, age, gender/gender, parent education, family structure, school achievement, and tobacco use susceptibility. Age was a dichotomous variable 0 for lower than 15 and 1 for 15 and above. Gender was 1 for male and 0 for female. School achievement was the independent variable, and tobacco use susceptibility was the outcome, both treated as continuous measures. 

*Tobacco use susceptibility.* Tobacco use susceptibility was self-reported and measured using the following indicators: (a) Think you will smoke a cigarette in the next year, (b) Think you will try a cigarette soon, and (c) Would smoke a cigarette if one of your best friends offered you one. Responses were between 1 (definitely no) to 4 (definitely yes). Total score ranged between 3 to 12. Reliability of this measure was 0.835.

*School Achievement.* Adolescents’ school achievement was measured by asking participants about their grades. The exact item was, “What is your current overall school achievement?” The possible answers were 9 = A (“93–100”), 8 = A− (“90–92”), 7 = B+ (“87–89”), 6 = B (“83–86”), 5 = B− (“80–82”), 4 = C+ (“77–79”), 3 = C (“73–76”), 2 = C − (“70–72”), and 1 = D (”69 or below”). School achievement was a continuous measure, with a potential range from 1 to 9. A higher score indicated better school achievement.

*Parent education.* Parent education was a five-level variable as below: 1 = “Some high school”, 2 = “Completed high school”, 3 = “Some college”, 4 = “Completed college”, 5 = “Graduate or professional school after college.” This variable was a nominal variable.

*Family Structure*. Family structure was a dichotomous variable that reflected married parents and any other conditions (divorced, not married, partnered, etc.). We coded married as 1 and other conditions as 0.

*Ethnicity.* Ethnicity was self-identified, treated as a nominal variable. Ethnicity was the moderator variable (Non-Latino White, Latino, and African American). 

### 2.4. Data Analysis

Data analysis was performed using SPSS 24. SPSS was used for univariate, bivariate, and multivariable analysis. Univariate was descriptive statistics such as mean (standard deviation (SD)) and frequency (%). Bivariate included Pearson correlation test, Chi square, and independent samples *t* test. Four linear regression models were applied for multivariable modeling. Outcome was tobacco use susceptibility score, predictor was school achievement, and moderator was ethnicity, and age, gender, and parent education were the covariates. *Model 1* and *Model 2* were run in the pooled sample. *Model 3*, *Model 4*, *Model 5*, *and Model 6* were performed in Non-Latino White, Non-Latino African American, Latino White, and Latino African American adolescents, respectively. *Model 1* did not have and *Model 2* had the interaction term between ethnicity and predictor variable. B, SE, 95% CI, and p were reported from each model.

### 2.5. Institutional Review Board (IRB) 

This study used publicly available PATH data. All data are fully de-identified. Thus, the study was not human subject research and exempt from full IRB review.

## 3. Results

A total number of 3636 adolescents entered our analysis. Descriptive data of the total sample are reported in Table 1. Ethnic minority adolescents had lower school achievement and parent education than Non-Latino White adolescents.

Table 2 presents the summary of linear regressions for *Model 1* and *Model 2* that were fitted to the pooled sample. As this model shows, higher school achievement was associated with a lower level of tobacco use susceptibility, however this association was where stronger for Non-Latino White than ethnic minority adolescents.

Table 3 presents the summary of linear regressions for *Model 3*, *Model 4*, *Model 5*, and *Model 6* that were fitted to Non-Latino White, Non-Latino African American, Latino White, and Latino African American adolescents, respectively. As these models show, higher school achievement was associated with a lower level of tobacco use susceptibility for Non-Latino White but not for ethnic minority adolescents.

## 4. Discussion

This study found an inverse association between school achievement and tobacco use susceptibility in the pooled sample of US adolescents. That means that adolescents who are successful academically report low level of openness to smoke in the future. However, this association was weaker for ethnic minorities than Non-Latino White adolescents, meaning that the returns of educational success in terms of tobacco protection is diminished for African American and Latino than Non-Latino White adolescents. Similar patterns were found for African American and Latino adolescents, reflecting that African American and Latino adolescents remain at risk of smoking in the future, regardless of their success at school.

Our observed inverse relationship between baseline academic achievement and subsequent smoking susceptibility in the entire sample was because our sample was predominantly Non-Latino White, which showed such effect. The inverse association between school achievement and tobacco use susceptibility in Non-Latino White sample is in line with the Jesser and Jesser theory [1]. According to this theory, problematic behaviors tend to covary, and each risk behavior increases the likelihood of other risk behaviors. However, the mechanisms behind the overlap between various risk behaviors such as aggression, drug use, tobacco use, school dropout, and even health problems are not fully known [1]. These are important because clustering of problem behaviors suggest that we should address multiple risk behaviors through same interventions, and multi-behavioral approach [42]. Group differences in this clustering, however, suggests that these multi-behavioral interventions may be less relevant to some sociodemographic groups. We found that interventions that simultaneously address school achievement and tobacco use susceptibility are more relevant to Non-Latino White adolescents, while separate interventions may be more relevant to ethnic minority adolescents. Thus, this study adds to what we know about how to tailor interventions based on ethnicity [43]. This is because such clustering of risk behaviors seems to be diminished for ethnic minority adolescents. 

Ethnic differences exist in clustering of problem behaviors such as school dropout, tobacco use, general behavior, and aggression [44]. The literature, however, is mixed on whether ethnic minorities show stronger or weaker clustering of various risk behaviors than Non-Latino White individuals [11,12,13]. However, we are not aware of any previous studies on ethnic variation in the association between school achievement and tobacco use susceptibility. 

Our second results align with what Fuller-Rowell has shown in this area [11,12,13]. His first study showed that ethnic minority adolescents pay greater social costs with academic success than Non-Latino Whites; however, this is seen in highly achieving schools with a smaller percentage of ethnic minorities students [14]. His second study also showed that effects of educational attainment on inflammation levels is weaker for ethnic minorities than for Non-Latino Whites [12]. 

Our second finding, which was on a weaker association between school achievement and tobacco use susceptibility in ethnic minorities than Non-Latino White adolescents, is in line with the marginalization-related diminished returns which suggests resources and assets may generate fewer economic, behavioral, developmental, and health outcomes for marginalized and racialized than Non-Latino White individuals. Sense of mastery [33], positive affect [34,35], happiness [36,37,38], and sense of health [45,46] may have stronger real life implications in terms of objective health, life expectancy, and physical health for Non-Latino Whites than African American and Latino adolescents. For ethnic minority populations, environments are difficult to control. For Non-Latino White individuals, environment is supportive of ambitions. In an easy to control environment for Whites and hard to control environment for African Americans and Latinos, higher levels of educational success are more productive for Whites than African American and Latino individuals. Environments are not conducive of efficacy, and can even be detrimental [39]. In one study, after controlling for other factors, high sense of perceived control was associated with lower not higher life expectancy [33]. The positive association between educational success is also associated with John Henryism for ethnic minority individuals [47,48,49,50,51]. Hudson studied about high costs of success of ethnic minority adolescents and young adults [52]. Differences in locus of control [53], with Non-Latino White individuals showing higher internal and ethnic minorities individuals showing higher external locus of control [53], may also explain why correlates of educational achievement may vary by group.

This study replicated the MDRs, this time for tobacco use susceptibility, which is in line with past research [40,41]. Previous MDRs work has shown that the link between SES and tobacco use is racialized [40,41]. This study showed that the link between educational success and tobacco use susceptibility is also racialized in the US. We explain this finding by worse schools and neighborhoods of highly ambitious ethnic minority adolescents compared to highly ambitious Non-Latino White adolescents [17]. Attending worse schools and residing in poor neighborhoods means challenges of experiences, education, and play of ethnic minority adolescents than Non-Latino White adolescents [15,16]. Educational success may also have different behavioral correlates because ethnic minority adolescents are discriminated at schools and neighborhoods at all environments [15,16] and this is even worse for high SES African American and Latino adolescents in high SES Non-Latino White schools and neighborhoods [19,20]. 

## 5. Implications

Our results are important because differential clustering has implications for combining programs for adolescents. Our results advocate for simultaneous addressing of multiple problems by the same interventions and programs for Non-Latino White adolescents. The observed weakened and reduced clustering between various problems suggests that tailored interventions might be less appropriate for African American and Latino adolescents. However, our results were only suggestive and we need future replications before any final conclusion. If our results are successfully replicated in the future, combined programs may be more relevant to Non-Latino White adolescents, while ethnic minority adolescents may benefit more from the separate programs that address one, not multiple problems. Any decision to separate programs for ethnic groups should be made with caution as such separate programs may have the unintended effect and segregate ethnic groups even more.

## 6. Limitations

Our study had a few limitations. First, this was a study with imbalanced sample size (larger n for Non-Latino White than ethnic minority adolescents). However, our main inference was based on pooled sample analysis with interaction rather than stratified models which have differential power. Our study also did not test gender differences within the relationship between school achievement and tobacco use susceptibility. Finally, all data were self-reported which was subject to social desirability and under-reporting. None of these issues are fatal flaws. A major contribution of this study is its longitudinal design. Other strengths included four years of follow up, the nationally representative sample, and the large sample size.

## 7. Conclusions

To conclude, we tested the association between baseline school achievement and subsequent tobacco use susceptibility of US adolescents overall and by ethnicity. Our first result showed that overall, higher level of school achievement is associated with lower level of tobacco use susceptibility in US adolescents. Our second finding showed that this inverse association is weaker for African American and Latino than Non-Latino White adolescents. A weaker protective effect of school achievement on tobacco use susceptibility of African American and Latino than non-Latino White adolescents may reflect structural inequalities in schools and neighborhoods of ethnic minorities communities.

## Figures and Tables

**Table 1:** Descriptive data overall in adolescents.

	All	
	*n*	%
Ethnicity		
Non-Latino Whites	2728	75.0
Latino	908	25.0
White	2872	79.0
African American	764	21.0
Age		
12–14	3511	96.6
15–18	125	3.4
Gender/Sex		
Male	1769	48.7
Female	1867	51.3
Marital Status of the Parents (Wave 4)		
Not Married	1250	34.4
Married	2386	65.6
Parental Education (1–5)	2.8861	1.25430
School Grades (Wave 1)	7.5215	1.52407
Subsequent Tobacco Use Susceptibility (Wave 4)	3.4359	0.99574

*p* < 0.05 for comparison of Non-Latino White, African American, and Latino adolescents.

**Table 2:** Pooled sample models in Non-Latino White and ethnic minority adolescents.

	Unstandardized B	Unstandardized Std. Error	Standardized Beta	Sig.	Lower Bound	Upper Bound
Model 1 (All, Main Effects)						
African American	−0.193	0.044	−0.079	0.000	−0.279	−0.107
Latino	0.035	0.042	0.015	0.395	−0.046	0.117
Male	−0.100	0.033	−0.050	0.003	−0.166	−0.035
Age (Wave 1)	−0.023	0.090	−0.004	0.802	−0.200	0.155
Married Parents (Wave 1)	0.011	0.037	0.005	0.761	−0.062	0.084
Parent Education (1–5) (Wave 1)	0.035	0.015	0.044	0.019	0.006	0.063
School Grades (1–9) (Wave 1)	−0.040	0.012	−0.061	0.001	−0.063	−0.017
Model 2 (All, M1 + Interaction)						
African American	−0.687	0.196	−0.281	0.000	−1.070	−0.303
Latino	−0.572	0.196	−0.249	0.004	−0.957	−0.188
Male	−0.102	0.033	−0.051	0.002	−0.167	−0.037
Age (Wave 1)	−0.019	0.091	−0.003	0.833	−0.197	0.158
Married Parents (Wave 1)	0.013	0.037	0.006	0.732	−0.060	0.086
Parent Education (1–5) (Wave 1)	0.039	0.015	0.049	0.008	0.010	0.068
School Grades (1–9) (Wave 1)	−0.078	0.016	−0.120	0.000	−0.109	−0.048
School Grades (1–9) (Wave 1) × African American	0.067	0.027	0.197	0.011	0.015	0.119
School Grades (1–9) (Wave 1) × Latino	0.081	0.026	0.266	0.002	0.031	0.132

Outcome: Tobacco use susceptibility Score (Wave 4); Data: Population Assessment of Tobacco and Health (PATH).

**Table 3:** Stratified models in African American, Latino, and Non-Latino White adolescents.

	Unstandardized B	Unstandardized Std. Error	Standardized Beta	Sig.	Lower Bound	Upper Bound
Model 3 (Non-Latino Whites)						
Male	−0.131	0.047	−0.062	0.005	−0.222	−0.039
Age (Wave 1)	0.113	0.128	0.019	0.381	−0.139	0.364
Married Parents (Wave 1)	0.031	0.056	0.012	0.581	−0.079	0.141
Parental Education (1–5) (Wave 1)	0.058	0.021	0.064	0.006	0.016	0.099
School Grades (1–9) (Wave 1)	−0.088	0.017	−0.120	0.000	−0.122	−0.054
Model 4 (Non-Latino African American)						
Male	−0.065	0.064	−0.040	0.313	−0.191	0.061
Age (Wave 1)	−0.164	0.137	−0.047	0.231	−0.432	0.104
Married Parents (Wave 1)	0.006	0.066	0.004	0.921	−0.123	0.136
Parental Education (1–5) (Wave 1)	0.027	0.028	0.039	0.323	−0.027	0.082
School Grades (1–9) (Wave 1)	−0.006	0.021	−0.011	0.783	−0.047	0.035
Model 5 (Latino White)						
Male	−0.078	0.071	−0.039	0.270	−0.216	0.061
Age (Wave 1)	−0.178	0.252	−0.025	0.481	−0.674	0.318
Married Parents (Wave 1)	−0.046	0.073	−0.022	0.525	−0.189	0.097
Parental Education (1–5) (Wave 1)	0.008	0.031	0.009	0.793	−0.053	0.069
School Grades (1–9) (Wave 1)	0.013	0.023	0.020	0.571	−0.033	0.059
Model 5 (Latino African American)						
Male	−0.062	0.202	−0.035	0.761	−0.464	0.341
Age (Wave 1)	−0.350	0.662	−0.060	0.599	−1.668	0.968
Married Parents (Wave 1)	0.201	0.214	0.108	0.350	−0.225	0.628
Parental Education (1–5) (Wave 1)	−0.019	0.085	−0.026	0.819	−0.188	0.149
School Grades (1–9) (Wave 1)	0.022	0.070	0.036	0.759	−0.118	0.161

Outcome: Tobacco use susceptibility Score (Wave 4); Data: Population Assessment of Tobacco and Health (PATH).

## Data Availability

This study used publicly available PATH data. All data are fully de-identified. PATH data are available here: https://www.icpsr.umich.edu/web/NAHDAP/series/606 (accessed on 20 January 2023).
